# Smoking cessation is associated with regression of electrocardiographic left ventricular hypertrophy: A 6-year retrospective observational study

**DOI:** 10.1371/journal.pone.0357409

**Published:** 2026-08-27

**Authors:** Yuichi Ninomiya, Shin Kawasoe, Takuro Kubozono, Eiko Sai, Norihito Nuruki, Hiroyuki Kamada, Yasuhisa Iriki, Yuichi Akasaki, Kunitsugu Takasaki, Tetsuro Kataoka, Hironori Miyahara, Koichi Tokushige, Mitsuru Ohishi

**Affiliations:** 1 Department of Cardiovascular Medicine, NHO Kagoshima Medical Center, Kagoshima, Japan; 2 Department of Cardiovascular Medicine and Hypertension, Kagoshima University Graduate School of Medical and Dental Sciences, Kagoshima, Japan; 3 Kagoshima Kouseiren Hospital, Kagoshima, Japan; Scuola Superiore Sant’Anna, ITALY

## Abstract

**Background:**

Electrocardiographic left ventricular hypertrophy is a known risk factor for cardiovascular morbidity and mortality. Although smoking cessation has general cardiovascular benefits, its impact on left ventricular hypertrophy regression remains unclear.

**Objective:**

To investigate whether smoking cessation is associated with regression of electrocardiographic left ventricular hypertrophy.

**Methods:**

We analyzed data from 752 non-hypertensive participants (695 males; mean age, 47.8 ± 9.2 years) who underwent annual health checkups. Left ventricular hypertrophy was defined using the Sokolow–Lyon criteria (SV1 + RV5 > 3.5 mV). Measurements were compared between baseline and 6 years after smoking cessation. Multivariable logistic regression analysis was used to identify predictors of left ventricular hypertrophy progression.

**Results:**

After 6 years, the average left ventricular voltage decreased significantly (2.47 → 2.41 mV, p < 0.0001), and the prevalence of left ventricular hypertrophy declined from 8.4% to 6.5%. This regression occurred despite increases in body weight (65.7 → 68.1 kg, p < 0.0001) and systolic blood pressure (112.8 → 122.0 mmHg, p < 0.0001). Left ventricular hypertrophy regression was observed in both participants who received antihypertensive medication and in those who did not. Multivariable analysis identified antihypertensive medication as an independent predictor of electrocardiographic LVH regression.

**Conclusions:**

Smoking cessation is associated with regression of electrocardiographic left ventricular hypertrophy, independent of weight gain or increases in blood pressure.

## Introduction

Left ventricular hypertrophy (LVH) is a well-established, independent risk factor for cardiovascular morbidity and mortality [1]. It reflects structural and functional adaptations of the myocardium in response to increased workload and neurohormonal stimulation. Electrocardiographic assessment of LVH remains a widely used and accessible method for cardiovascular risk stratification, despite some limitations in sensitivity compared to imaging-based modalities [2]. Numerous longitudinal studies have demonstrated that regression of electrocardiographic LVH is associated with improved cardiovascular outcomes, including reduced incidence of stroke, heart failure, and sudden cardiac death [3,4].

Cigarette smoking is a well-documented, modifiable risk factor for cardiovascular disease. In Japan, smoking rates are declining in both males and females, although the rate of decline tends to be greater among males [5]. Chronic tobacco exposure contributes to endothelial dysfunction, increased sympathetic tone, arterial stiffness, and adverse cardiac remodeling [6–8]. Although the vascular and metabolic benefits of smoking cessation are well recognized [8], the extent to which cessation reverses structural cardiac changes, such as LVH, is not fully understood. Several studies have shown that current smokers exhibit greater left ventricular mass and wall thickness than non-smokers [9–11]. However, longitudinal data examining whether smoking cessation leads to regression of LVH remain limited. Understanding this potential relationship is crucial, as it may reinforce the structural and prognostic benefits of smoking cessation beyond risk factor modification.

In this study, we aimed to evaluate the association between smoking cessation and changes in electrocardiographic LVH over a 6-year period in a Japanese population cohort undergoing regular health examinations. We hypothesized that smoking cessation would lead to a significant regression of electrocardiographic LVH, even in the presence of potential confounding factors such as weight gain and elevated blood pressure.

## Methods

### Study population and design

**Fig 1:**
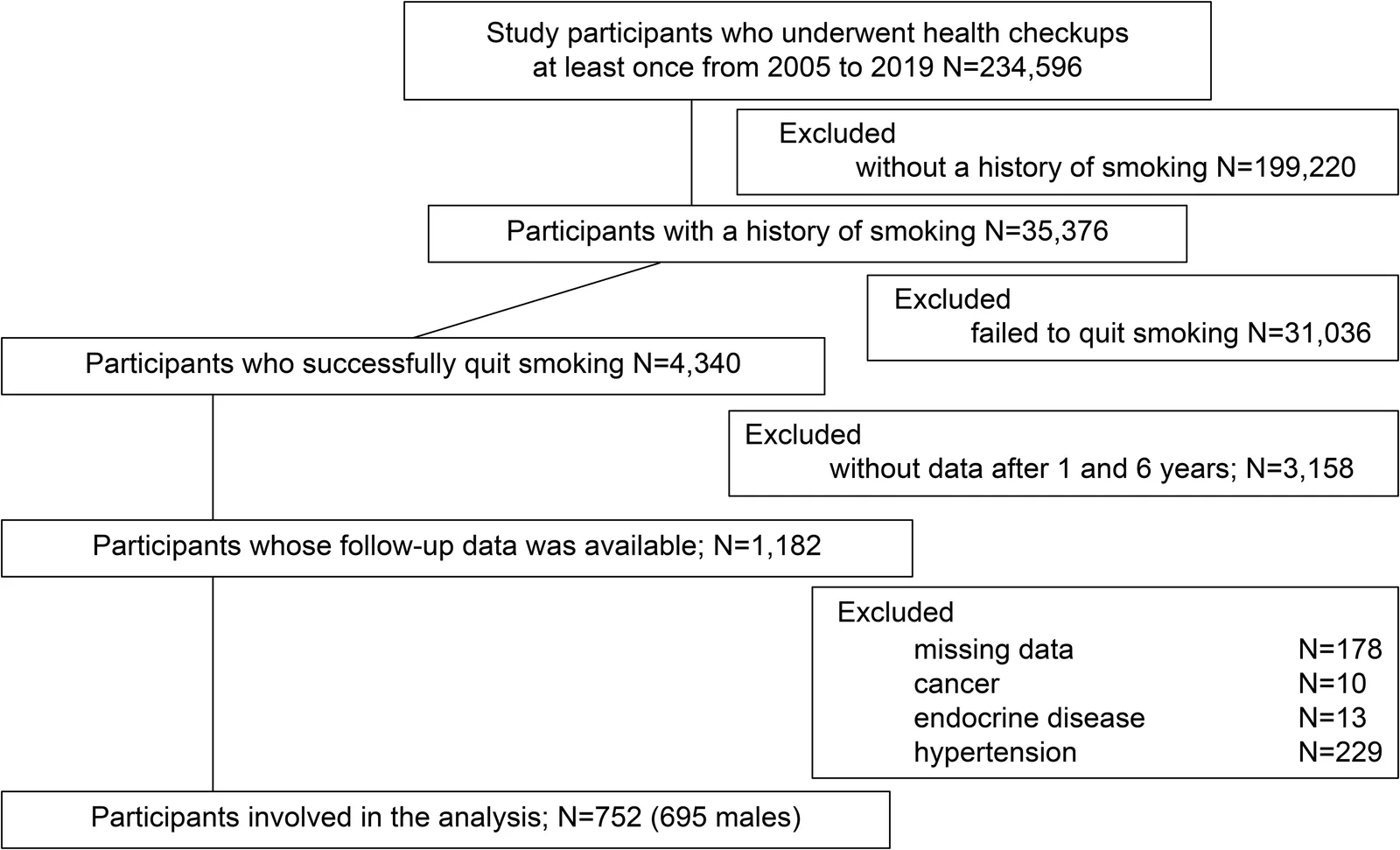
Flowchart showing the study participant enrollment process.

This retrospective observational study was conducted using a large-scale dataset comprising 234,596 health examination records from the general Japanese population, as described previously [12]. We included participants who underwent at least one health examination between 2005 and 2019. From this cohort, we selected 752 participants who had quit smoking and were free of hypertension at baseline. The inclusion criteria were (1) male and female participants aged 30–65 years, (2) no diagnosis of, or treatment for, hypertension at the time of smoking cessation, and (3) availability of electrocardiogram (ECG) data both at baseline and 6 years after cessation. Participants who were current smokers or had co-morbidities, including cancer, endocrine disorders, or hypertension (defined as SBP ≥ 140 mmHg and/or DBP ≥ 90 mmHg or antihypertensive drug use at baseline), as well as those with missing ECG data, were excluded. Participants with structural heart disease, a history of myocardial infarction, or incomplete follow-up data were also excluded (Fig 1).

### Electrocardiographic assessment of LVH

Standard 12-lead ECGs were recorded annually using automated recording systems. We used automatic measurements and interpretations of 12-lead ECGs. Heart rate, QRS duration, axis range, QT interval, SV1, and RV5 were obtained from the automated ECG measurement. Electrocardiographic LVH was defined using the Sokolow–Lyon voltage criterion (SV1 + RV5 > 3.5 mV). Although less sensitive than echocardiographic assessment, this method has been shown to be a reliable surrogate marker in population-based studies and correlates well with cardiovascular outcomes [13].

### Clinical measurements

Baseline and follow-up data included body weight, body mass index (BMI), systolic and diastolic blood pressure, lipid profiles (low-density lipoprotein cholesterol and high-density lipoprotein cholesterol), and estimated glomerular filtration rate (eGFR). eGFR was calculated using the following formula: eGFR (mL/min/1.73 m^2^) = 194 × serum creatinine − 1.094 × age − 0.287 × 0.739 (female) [14]. An eGFR < 60 mL/min/1.73 m^2^ indicated chronic kidney disease. All measurements were performed according to standardized protocols. Cardiovascular risk factors included the following: diabetes, defined as fasting blood glucose level ≥126 mg/dL or active treatment with oral hypoglycemic agents or insulin; and dyslipidemia, defined as serum triglyceride level ≥150 mg/dL, serum low-density lipoprotein cholesterol level ≥140 mg/dL, serum high-density lipoprotein cholesterol level <40 mg/dL, or use of lipid-lowering agents [15]. Use of antihypertensive medication during the follow-up period was also recorded. Participants were divided into two groups based on whether they received antihypertensive medications during the follow-up. We compared baseline characteristics between the two groups.

### Ethics approval

The Institutional Ethics Committee of the Kagoshima University Graduate School of Medical and Dental Sciences approved this retrospective study (approval no. 170130[520], December 13, 2021). The requirement for informed consent was waived because anonymized data were analyzed. The study protocol was in accordance with the principles of the Declaration of Helsinki. The authors had no access to information that could identify individual participants during or after data collection. The dataset was accessed on 03/02/2024.

### Statistical analysis

Continuous variables were expressed as mean ± standard deviation, except for triglyceride levels, which were expressed as median (first quartile–third quartile). Categorical variables were expressed as absolute numbers and percentages. Baseline characteristics were compared between participants who received antihypertensive medications during follow-up and those who did not. Changes in continuous variables between baseline and the 6-year follow-up were analyzed using paired t-tests. The proportions of participants with electrocardiographic LVH at the two time points were compared using McNemar’s test. Multivariable logistic regression was employed to identify independent predictors of electrocardiographic LVH progression, adjusting for confounding factors such as age, BMI change, systolic blood pressure change, smoking history, and medication use. A p-value of < 0.05 was considered statistically significant. All statistical analyses were conducted using JMP Pro software, version 16.0 (SAS Institute Inc., Cary, NC, USA).

## Results

### Participant characteristics

Of the 752 participants included in the final analysis, 695 (92.4%) were male. The mean baseline age was 47.8 ± 9.2 years. Baseline systolic blood pressure was 112.8 ± 12.7 mmHg, and the mean BMI was 23.3 ± 2.8 kg/m². The mean low-density lipoprotein cholesterol was 120.4 ± 28.1 mg/dL, the high-density lipoprotein cholesterol was 58.1 ± 13.6 mg/dL, and the eGFR was 80.2 ± 13.3 mL/min/1.73 m². None of the participants had been treated with antihypertensive medications at baseline (Table 1).

**Table 1:** Baseline characteristics of the participants.

	All	Administered	Non-administered	P-value
	(n = 752)	(n = 103)	(n = 649)	(Ad vs. Non-ad)
Age (years)	47.8 ± 9.2	53.5 ± 7.8	46.9 ± 9.1	<0.001
Sex (Male)	695 (92.4)	102 (99.0)	593 (91.4)	<0.01
Body weight (kg)	65.7 ± 10.5	66.0 ± 9.8	65.7 ± 10.7	0.7587
BMI (kg/m2)	23.2 ± 3.0	23.6 ± 3.0	23.1 ± 3.0	0.1187
Habitual drinking	423 (56.3)	76 (73.8)	347 (53.5)	<0.001
SBP (mmHg)	112.8 ± 12.7	124.1 ± 9.6	111.1 ± 12.2	<0.001
DBP (mmHg)	72.1 ± 9.2	79.0 ± 7.6	71.0 ± 8.9	<0.001
Diabetes	44 (5.9)	13 (12.6)	31 (4.8)	<0.01
Dyslipidemia	379 (50.4)	56 (54.4)	323 (49.8)	0.3857
Total cholesterol (mg/dL)	202.5 ± 32.9	207.9 ± 33.9	201.7 ± 32.7	0.0813
Triglyceride (mg/dL)	106 (75, 161)	107 (80, 183)	105 (73, 160)	0.1355
LDL-C (mg/dL)	121.4 ± 30.1	123.9 ± 29.8	121.0 ± 30.1	0.3573
HDL-C (mg/dL)	54.2 ± 13.8	55.2 ± 13.8	54.0 ± 13.8	0.4425
eGFR (mL/min/1.73m^2^)	81.5 ± 13.3	78.8 ± 13.2	81.9 ± 13.2	<0.05
SV1 + RV5 (mV)	2.47 ± 0.70	2.68 ± 0.74	2.44 ± 0.69	<0.01

Values are presented as the mean±standard deviation or n (%). BMI, body mass index; SBP, systolic blood pressure; DBP, diastolic blood pressure; LDL-C, low-density lipoprotein cholesterol; HDL-C, high-density lipoprotein cholesterol; eGFR, estimated glomerular filtration rate.

### Changes in body weight and blood pressure

After 6 years of smoking cessation, participants experienced a significant increase in mean body weight from 65.7 ± 10.5 kg to 68.1 ± 10.8 kg (p < 0.0001). Similarly, systolic blood pressure increased significantly from 112.8 ± 12.7 mmHg to 122.0 ± 14.2 mmHg (p < 0.0001). A total of 103 participants (13.7%) required initiation of antihypertensive medication during the 6-year follow-up. Therefore, as described in the Methods section, participants were divided into two groups based on whether they received antihypertensive medications during the 6-year follow-up. In the group receiving antihypertensive medications, significant differences were observed in the following factors: higher age, greater proportions of males and habitual drinkers, higher systolic and diastolic blood pressure, higher prevalence of diabetes, lower eGFR, and higher LV voltage at baseline (Table 1).

### Regression of electrocardiographic LVH

**Fig 2:**
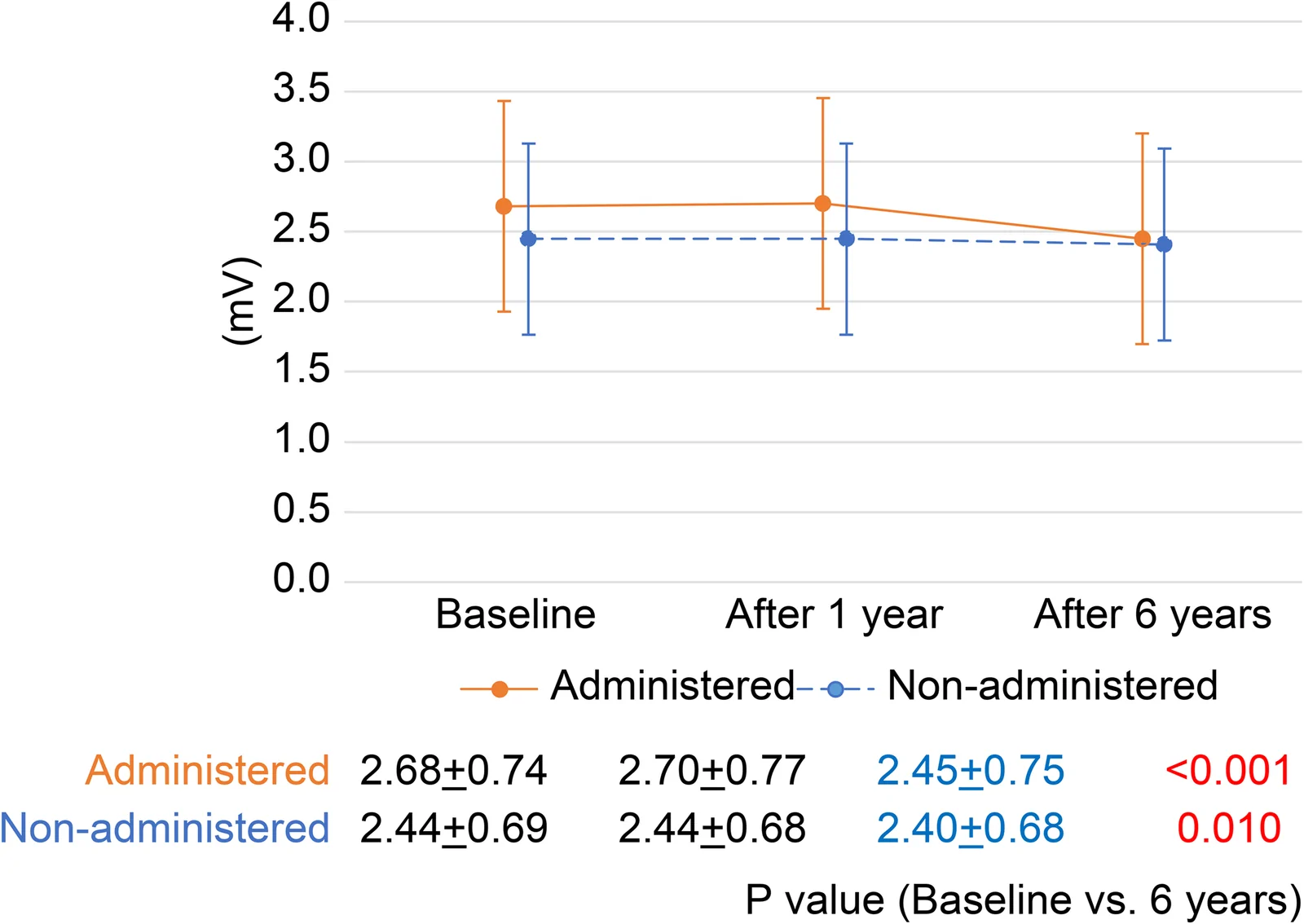
Regression of electrocardiographic LVH after smoking cessation.

The mean LV voltage (SV1 + RV5) decreased significantly from 2.47 mV to 2.41 mV (p < 0.0001) over the 6-year period. The prevalence of ECG-defined LVH (SV1 + RV5 > 3.5 mV) declined from 8.4% to 6.5%. When stratified by antihypertensive medication status, both groups showed significant reductions in LV voltage (Fig 2):

- With antihypertensive medications: 2.68 → 2.45 mV (p < 0.0001)
- Without antihypertensive medications: 2.44 → 2.40 mV (p = 0.01)

p-value: baseline vs. 6 years

### Predictors of the progression of electrocardiographic LVH

The multivariable analysis included general factors associated with the progression of LVH and factors that differed between the patient characteristics. Multivariable analysis identified antihypertensive medication as an independent predictor of electrocardiographic LVH regression. (Table 2).

**Table 2:** Predictors of the progression of electrocardiographic LVH.

	Univariable analysis		Multivariable analysis	
	HR (95% CI)	p-value	HR (95% CI)	p-value
Age (years)	1.01 (0.99–1.03)	0.102	1.02 (1.00–1.03)	0.107
Sex (Male)	0.74 (0.43–1.27)	0.269	0.68 (0.38–1.20)	0.181
Antihypertensive Medications after 6 years	0.57 (0.37–0.88)	0.010	0.52 (0.33–0.82)	0.005
Weight gain after 1 year post-cessation (kg)	1.44 (0.40–5.12)	0.573	1.00 (0.95–1.06)	0.886
Diabetes	0.60 (0.32–1.14)	0.110	0.60 (0.31–1.15)	0.125
CKD	0.99 (0.42–2.33)	0.988	0.86 (0.35–2.11)	0.742
Habitual drinking	0.98 (0.73–1.31)	0.890	0.99 (0.73–1.34)	0.940
A 20 pack-year smoking history or more	1.32 (0.99–1.77)	0.059	1.34 (0.95–1.89)	0.095

CI, confidence interval; CKD, chronic kidney disease; HR, hazard ratio.

## Discussion

This study provides novel evidence that smoking cessation is associated with a modest but statistically significant regression of electrocardiographic LVH over a 6-year period. We previously reported that smoking cessation leads to weight gain and that greater weight gain is associated with a higher future risk of elevated blood pressure [12]. This effect was observed despite parallel increases in body weight and blood pressure, which are typically associated with adverse cardiac remodeling. These findings suggest that smoking cessation may confer direct or indirect benefits on left ventricular structure beyond traditional risk factor modification. Furthermore, antihypertensive medication was an independent predictor of electrocardiographic LVH regression. Based on these findings, patients should be advised to monitor for weight gain after smoking cessation, and antihypertensive medication should be initiated promptly if blood pressure increases following cessation.

The regression of electrocardiographic LVH observed in our cohort may be explained by several mechanisms. Smoking increases sympathetic nervous system activity and arterial stiffness, both of which impose pressure overload on the left ventricle. Smoking cessation may reverse these hemodynamic stresses, leading to favorable myocardial remodeling [16]. Additionally, improved endothelial function and reduced oxidative stress after smoking cessation may contribute to the attenuation of hypertrophic signaling pathways [17].

Previous findings from our cohort indicated that weight gain after smoking cessation is associated with future increases in blood pressure [12]. A notable finding of this study is that LVH regression occurred even among participants who experienced weight gain and systolic blood pressure increases in both the medicated and non-medicated subgroups. This supports the hypothesis that the removal of tobacco-related toxic effects may outweigh the adverse impact of modest metabolic deterioration post-cessation.

Our findings align with prior studies showing increased LV mass and wall thickness in active smokers, as well as reports indicating reversibility of smoking-induced vascular changes [18]. However, few studies have specifically examined longitudinal changes in electrocardiographic LVH in relation to smoking cessation, highlighting the unique contribution of our study.

Certain limitations should be acknowledged. First, ECG criteria, which are practical for large studies, may underestimate true LV mass when compared with echocardiography or cardiac MRI. Larger imaging-based studies, including those using echocardiography or cardiac MRI, are required to validate these findings and determine whether reverse structural remodeling after smoking cessation translates into improved clinical outcomes. In addition, changes in ECG voltage may partly reflect attenuation of the ECG signal due to increased adiposity rather than true regression of myocardial mass. Second, the predominantly male composition of this cohort may limit the generalizability of the findings. In Japan, this is thought to be attributable to the low smoking rate among females [5]. Other factors such as sex differences may influence electrocardiographic LVH; a previous report indicates that the accuracy of electrocardiographic diagnostic criteria for LVH is lower in females [19]. Therefore, the results should be interpreted with caution. Third, while we adjusted for major factors, residual confounding due to unmeasured lifestyle or socioeconomic factors cannot be excluded. Smoking cessation may be a marker of broader lifestyle changes rather than an isolated exposure. Finally, the observed changes were statistically meaningful but relatively small, and further studies are needed to elucidate their clinical impact.

## Conclusion

In this large-scale observational study, we found that smoking cessation was associated with significant regression of electrocardiographic LVH over 6 years, even in the presence of weight gain and elevated blood pressure. These findings reinforce the cardiovascular benefits of smoking cessation and support its role in reversing subclinical target organ damage. Clinicians should emphasize not only the long-term risk reduction associated with smoking cessation but also its potential to reverse existing cardiac remodeling.
